# Burdens of neck pain in China from 1990 to 2021 and its projection to 2050: Findings from the Global Burden of Disease study 2021

**DOI:** 10.12669/pjms.42.2.14050

**Published:** 2026-02

**Authors:** Guanghai Zhao, Xian Zhang, Xinrui Zhao, Shanming Zhang, Haihong Zhang

**Affiliations:** 1 Guanghai Zhao Department of Orthopaedics, The Second Hospital & Clinical Medical School, Lanzhou University, Lanzhou, Gansu Province 730000, P.R. China; 2 Xian Zhang Department of Orthopaedics, Liangzhou Hospital of Integrated Traditional Chinese and Western Medicine, Wuwei, Gansu Province 733000, P.R. China; 3 Xinrui Zhao Department of Orthopaedics, The Second Hospital & Clinical Medical School, Lanzhou University, Lanzhou, Gansu Province 730000, P.R. China; 4 Shanming Zhang Cuiying Honors College, Lanzhou University, Lanzhou, Gansu Province 730000, P.R. China; 5 Haihong Zhang Department of Orthopaedics, The Second Hospital & Clinical Medical School, Lanzhou University, Lanzhou, Gansu Province 730000, P.R. China

**Keywords:** Neck pain, Global burden of diseases, Injuries and risk factors study 2021, China

## Abstract

**Background & Objective:**

This study aimed to assess the burden of neck pain from 1990 to 2021 and predict its trends over the next three decades, providing evidence for targeted interventions.

**Methodology:**

This study is a secondary analysis of publicly available national-level estimates for China from the Global Burden of Disease (GBD) 2021 study released by the Institute for Health Metrics and Evaluation, covering 1990–2021, with projections to 2050. The age-standardized incidence rate (ASIR), age-standardized prevalence rate (ASPR), and age-standardized disability-adjusted life years (DALYs) rate for neck pain were calculated. Temporal trends were evaluated using estimated annual percentage changes (EAPC). The age-period-cohort (APC) model was employed to explore underlying drivers of disease burden, while the Bayesian-APC (BAPC) model was applied to predict future trends.

**Results:**

In 2021, the ASIR, ASPR, and age-standardized DALYs rate for neck pain in China were 567.23 (95% uncertainty interval [UI]: 448.497 – 699.796, per 100000 population), 2549.87 (95% UI: 2007.887 – 3141.637, per 100000 population), and 254.77 (95% UI: 166.889 – 357.934, per 100000 population), respectively. Between 1990 and 2021, the EAPCs for ASIR, ASPR, and age-standardized DALYs rates were 0.08% (95%CI: 0.06 – 0.11), 0.13% (95%CI: 0.10 – 0.16), and 0.13% (95%CI: 0.10 – 0.16), respectively. APC analysis identified age as the dominant contributing factor, with the highest burden observed in the 70 – 74-year age group and a consistently greater burden among females. Projections from the BAPC model suggest that ASIR, ASPR, and age-standardized DALYs rates will decline by 2025, although sex-based differences are expected to persist.

**Conclusion:**

Neck pain remains a significant public health concern in China, particularly among elderly individuals and females.

## INTRODUCTION

Neck pain, a common musculoskeletal condition with high prevalence and substantial disability, is recognized by the World Health Organization (WHO) as a significant global public health issue.^1^ Clinically, neck pain presents with localized discomfort and restricted mobility and is strongly associated with comorbidities such as headache, sleep disturbances, anxiety, depression, lower functional capacity and poor quality of life.^2^ The global burden of neck pain has been increasing, potentially driven by population aging, more sedentary occupational patterns, and greater reliance on electronic devices. According to the Global Burden of Disease Study (GBD) 2021,^3^ neck pain ranked 11th among 369 diseases in terms of years lived with disability (YLDs), underscoring its non-fatal yet significant impact on health.^4^ From a socioeconomic perspective, both direct and indirect neck pain costs are considerable. Direct medical expenses include outpatient services, imaging, medications, and rehabilitation, whereas indirect costs stem from reduced productivity, absenteeism, and premature retirement.^5^ For instance, in the United States, total medical costs related to neck pain and low back pain reached 13.45 billion USD in 2016, ranking first among 154 diseases.^6^ This economic burden is especially significant in low- and middle-income countries (LMICs), where limited occupational health protections increase the impact on manual laborers and industrial workers.

The burden of neck pain in China has increased more rapidly than the average trends observed in Asia and globally.^7^ Contributing factors include occupational influences such as prolonged desk work and extensive use of electronic devices, accelerated population aging, and unhealthy lifestyle behaviors like inadequate physical activity. The COVID-19 pandemic may have further intensified this upward trend.^8^ Pandemic-related lifestyle adaptations, including remote work and online education, were associated with prolonged poor posture and increased static loading of the neck musculature, potentially triggering or aggravating neck pain. Moreover, psychological stressors associated with COVID-19, such as anxiety and depression, may modulate pain perception through neurophysiological and psychosocial pathways, thereby exacerbating neck pain symptoms.^9^

Despite its growing impact, few studies have examined the burden of neck pain in China, and comprehensive, long-term nationwide epidemiological data remain limited. This gap significantly hinders the development and implementation of effective prevention and control strategies. Using the newly updated GBD 2021 estimates, we analyzed the incidence, prevalence, and age-standardized disability-adjusted life years (DALYs) rate of neck pain in China from 1990 to 2021. We further quantified long-term trends using EAPC, disentangled age, period, and cohort effects using an APC framework, and generated projections to 2050 using Bayesian age-period-cohort (BAPC) modeling, with detailed age- and sex-specific characterization to inform prevention and management strategies.

To our knowledge, this study adds to the existing literature by providing an up-to-date assessment based on GBD 2021, integrating complementary approaches (EAPC, APC, and BAPC) within one unified framework to move beyond descriptive trends toward interpretable mechanisms and projections, and offering refined age- and sex-specific insights that enhance the policy and clinical relevance of the findings.

## METHODOLOGY

This study is a secondary analysis of publicly available national-level estimates for China from the Global Burden of Disease (GBD) 2021 study released by the Institute for Health Metrics and Evaluation. We assessed neck pain burden from 1990 to 2021 and projected trends to 2050. As the most extensive and comprehensive epidemiological study to date, GBD 2021 evaluated age- and sex-specific incidence, prevalence, mortality, years of life lost (YLLs), YLDs, and DALYs for 369 diseases and injuries across 204 countries and territories from 1990 to 2021.^10^ Among these metrics, DALYs serve as a critical measure of disease burden, integrating the effects of both YLLs and YLDs to quantify the overall impact of diseases on population health.^11^

The definition of neck pain used in this study follows the standard adopted by GBD 2021: symptomatic neck pain lasting for at least one month within the past 12 months.^12^ Importantly, as the GBD study found no evidence of mortality attributable to neck pain, DALYs are equivalent to YLDs for this condition.^13^ Data were extracted from the official GBD website using “Neck pain” as the search term. The methodologies employed in GBD 2021, along with enhancements made relative to previous iterations, have been comprehensively described in earlier publications.^10^,^14^

GBD 2021 estimates for non-fatal conditions are generated through a standardized Bayesian meta-regression framework (DisMod-MR 2.1). For neck pain, DisMod-MR 2.1 fits age-, sex-, and year-specific incidence and prevalence using a hierarchical cascade (global–super-region–region–country), borrowing strength from higher levels when local data are sparse. Missing or sparse observations are implicitly handled by combining available data with covariates and location-specific random effects to produce smoothed and internally consistent estimates across adjacent ages, years, and locations. Uncertainty due to data sparsity and modeling assumptions is propagated to all outputs and reported as 95% uncertainty intervals (UIs).

### Age-standardized rate (ASR):

ASR was used to describe epidemiological indicators related to neck pain, including the age-standardized incidence rate (ASIR), age-standardized prevalence rate (ASPR), and age-standardized DALYs rate.^15^ ASRs were used to minimize the influence of changes in population age structure, whereas changes in absolute numbers may reflect population growth and population aging in addition to changes in age-specific rates. The estimation and analysis of these indicators were performed within the GBD database framework using the Bayesian regression tool DisMod-MR 2.1:


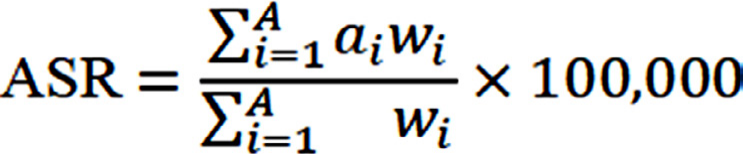


where α_i_ refers to the percent of the *i*th age group, ω_i_ refers to the population weight of the corresponding age group in the world standard population, and A refers to the total number of age groups. In terms of uncertainty quantification, this study conducted 500 repeated samplings for each indicator using a Monte Carlo simulation, which generated 500 parameter estimates. The final estimates were derived from the median of all sampling results, with the 95% UI defined by the 2.5th and 97.5th percentiles.

To evaluate the long-term trend of the disease burden, the study applied the Estimated Annual Percentage Change (EAPC).^16^ A linear regression model was established to examine the relationship between time and the natural logarithm of the ASR (ln[ASR]):Y = α + βx + ε. Where Y denotes ln (ASR), x denotes the calendar year, and ε denotes the error term. EAPC was calculated as: EAPC=100× [exp (β) - 1], with its 95% confidence interval (CI) derived from the regression model. A significantly increasing trend was defined as both the EAPC and the lower bound of its 95% CI being greater than zero. Conversely, a significantly decreasing trend was defined as both the EAPC and the upper bound of its 95% CI is less than zero. Otherwise, the trend was considered stable.

### APC model:

To analyze the long-term trends and underlying factors influencing the burden of neck pain in China, an APC model was employed to separate the independent effects of age, period, and birth cohort. The model can be expressed as follows:


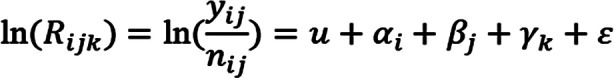


where *R_ijk_* refers to the incidence rate, prevalence rate, or DALYs rate of neck pain of the *i*th age group in the *k*th birth cohort during the *j*th period, *y_ij_* and *n_ij_* refer to the number of events and the exposed population, respectively, *u* refers to the intercept term, *α_i_*, *β_j_*, and *γ_k_* refer to the age, period, and cohort effects, respectively, and *ε* refers to the random error term.

In this study, neck pain data from China between 1990 and 2021 were stratified into five-year age groups and analyzed using the R-language APC toolkit developed by IHME.^17^ Because age, period, and cohort are exactly linearly dependent (cohort = period − age), APC parameters are not uniquely identifiable without constraints; therefore, we adopted the intrinsic estimator (IE), which yields a unique and stable solution that is relatively insensitive to the choice of reference categories and is widely used for population-level trend decomposition. The middle age group, period, and cohort were set as reference categories with a relative risk (RR) of 1.0. The model estimated several key parameters: net drift (overall annual percentage change across periods and cohorts), local drift (age-specific annual percentage change), and age effects (age-specific rates adjusted for period deviations with the reference cohort fixed). Period effects represent age-specific rates adjusted for cohort effects using the reference period, and cohort effects quantify relative risks for each birth cohort compared with the reference cohort after adjusting for age and non-linear cohort deviations. Model adequacy was examined by comparing fitted and observed age-specific rates and by inspecting deviance residuals for systematic patterns.

Statistical significance was evaluated using the Wald chi-square test (two-sided, α = 0.05). 95% CI were derived through bootstrapping with 1000 resamples. Model robustness was confirmed by sensitivity analyses, such as altering the reference group. To address data heterogeneity and model uncertainty, 95% uncertainty intervals (UIs) are presented.

### BAPC model:

Future trends in the burden of neck pain in China were projected using a Bayesian age–period–cohort (BAPC) model fitted via Integrated Nested Laplace Approximation (INLA).^18^ Following the BAPC package specification, age, period, and cohort effects were modeled as second-order random walks (RW2) (scale.model = FALSE), and an additional independent and identically distributed (iid) random effect was included to account for overdispersion. The (log) precision parameters of the RW2 components were assigned log-gamma priors (shape = 1, rate = 0.00005) with an initial value of 4 on the log-scale (default settings), and the overdispersion term used a log-gamma prior (shape = 1, rate = 0.005) with an initial value of 4. Projections to 2050 were generated using the predict option in BAPC, and results are reported as posterior medians with 95% uncertainty intervals (2.5th–97.5th percentiles). Model fit was assessed using INLA-based criteria (DIC/WAIC) and fitted-versus-observed comparisons; sensitivity checks using alternative smoothing (RW1) and alternative prior specifications did not materially change the projected patterns. All analyses were performed in R (Version 4.2.2), with data preprocessing and model fitting conducted using the WH Health Equity Assessment Toolkit, and visualizations generated with ggplot2 (Version 3.5.1).^19^

## RESULTS

**Fig.1:**
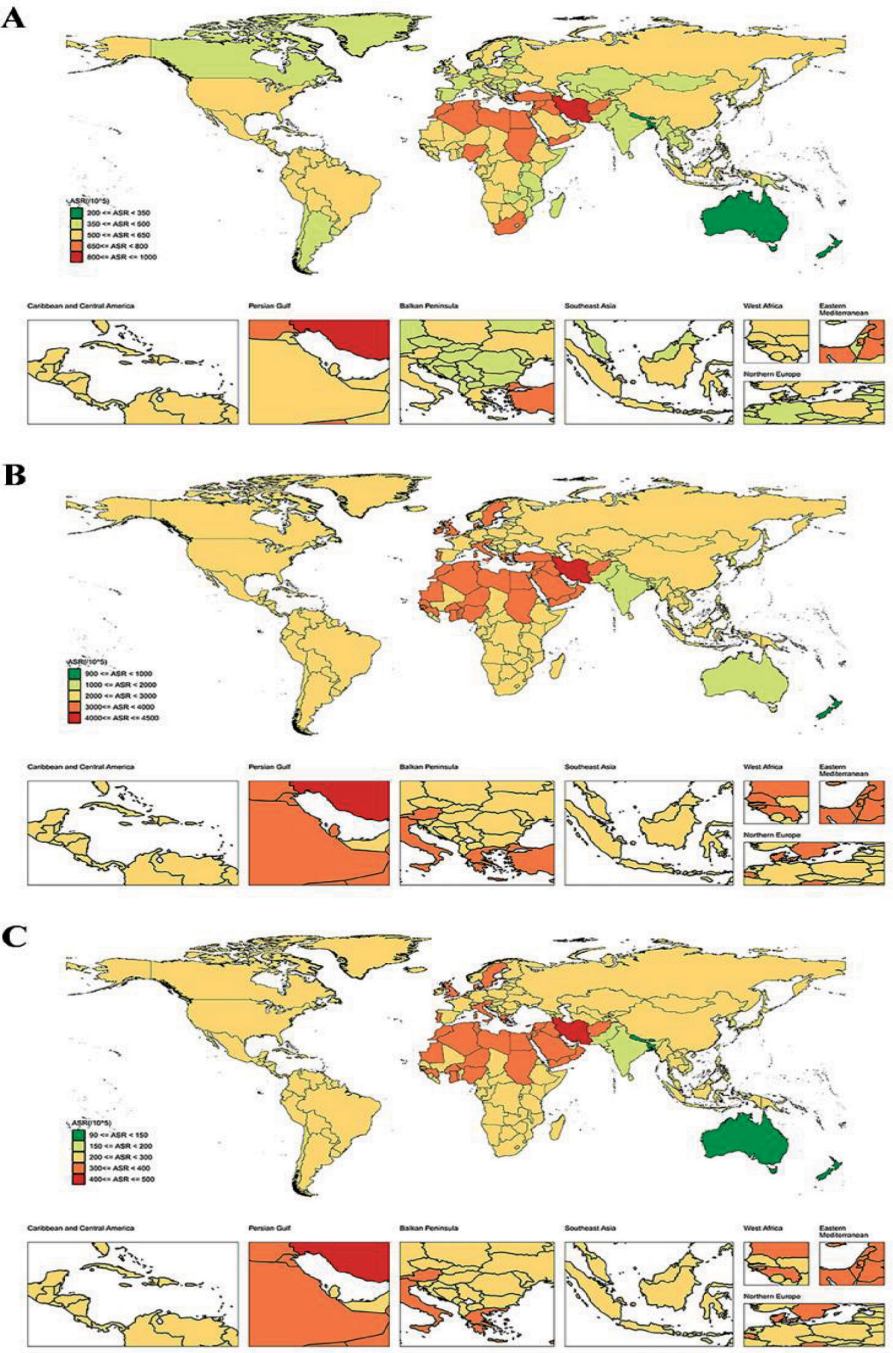
Global burden of neck pain, 2021. (A) ASIR; (B) ASPR; (C) Age-standardized DALYs rate.

**Fig.2:**
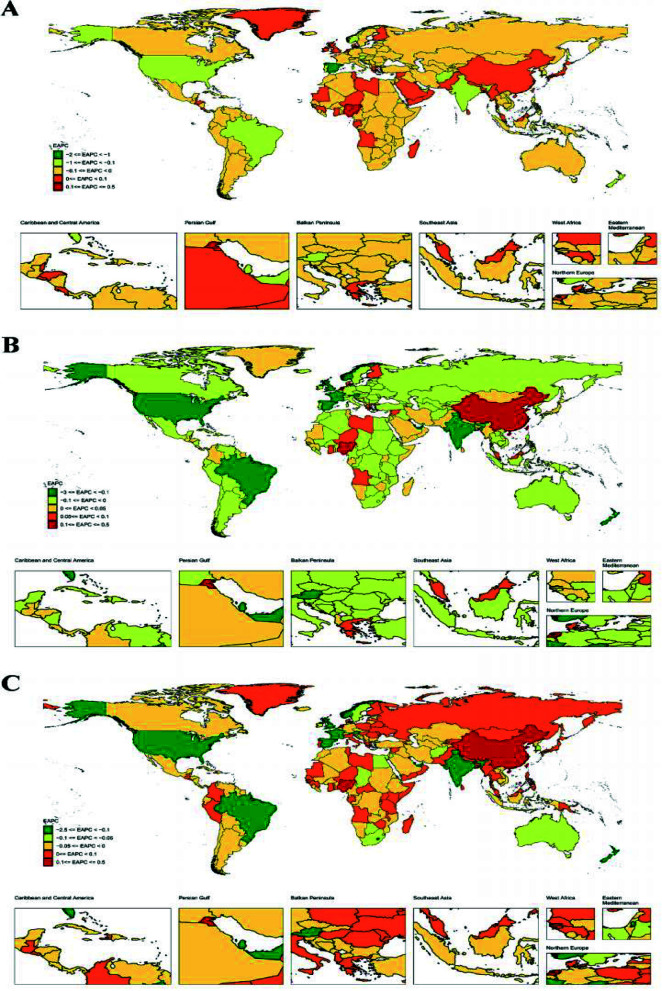
Trends in the global burden of neck pain, 1990-2021. (A) ASIR; (B) ASPR; (C) Age-standardized DALYs rate.

The geographic distribution of ASIR, ASPR and age-standardized DALYs rate for neck pain worldwide. Fig.1. Trends in the global burden of neck pain, 1990-2021, are shown in Fig.2.

In 2021, the ASIR of neck pain in China was 567.227 (95% UI: 448.497 – 699.796, per 100,000 population), significantly exceeding that of other global regions excluding Africa, which was 602.372 (95% UI: 469.983 – 742.432, per 100,000 population) (Fig.1A). Incidence increased from 6,192,332 (95% UI: 4,778,922 – 7,748,853) in 1990 to 10,292,099 (95% UI: 8,062,751 – 13,039,595) in 2021, representing a 66.2% rise (Table-I). From 1990 to 2021, the EAPC of the ASIR in China was 0.08 (95% CI: 0.06 – 0.11), significantly higher than that observed across five continents and neighboring countries such as North Korea, South Korea, Japan, and India (Fig.2A).

**Table-I:** Incidence, prevalence and DALYs of neck pain in China in 1990 and 2021.

	Year	Male	Female	Both
Incidence	1990	2786587 (2139157-3487342)	3405745 (2615619-4251514)	6192332 (4778922-7748853)
	2021	4481219 (3481378-5687594)	5810880 (4580452-7341714)	10292099 (8062751-13039595)
Prevalence	1990	11644289 (8887146-14697208)	14885766 (11515444-18433544)	26530055 (20347966-32917309)
	2021	20100389 (15686712-25117319)	28277014 (21872003-35243386)	48377404 (37665091-60063296)
DALYs	1990	1184796 (774026-1719943)	1490376 (977311-2174461)	1184796 (774026-1719943)
	2021	2013691 (1329396-2863369)	2793903 (1844449-3931334)	2013691 (1329396-2863369)

In 2021, the prevalence of neck pain in China reached 48,377,404 cases (95% UI: 37,665,091 – 60,063,296), reflecting an 82.3% increase since 1990 (Table-I). The ASPR of neck pain in China was 2,549.871(95% UI: 2,007.887 – 3,141.637, per 100000 population), ranking third after Europe (2,972.643 [95% UI: 2,315.282 – 3,683.911] per 100000 population) and Africa (2,871.467 [95% UI: 2,241.469 – 3,521.915] per 100,000 population), but significantly higher than other continents and neighboring countries (Fig.1B). The EAPC of the ASPR in China was 0.13 (95% CI: 0.10 – 0.16), significantly exceeding rates observed in five continents and neighboring countries, indicating a rapid increase in neck pain risk (Fig.2B).

Disease burden analysis indicated that the age-standardized DALYs rate for neck pain in China in 2021 was 254.774 (95% UI: 166.889 – 357.934), ranking third after Europe (285.589 [95% UI: 189.978 – 406.770] per 100000 population) and Africa (293.069 [95% UI: 195.104 – 417.375] per 100,000 population) (Fig.1C), with a total DALYs of 2,013,691 (95% UI: 1,329,396 – 2,863,369) (Table-I). Since 1990, age-standardized DALYs rates have shown a significant upward trend (EAPC = 0.13 [95% CI: 0.10 – 0.16]), with an increasing rate that is significantly higher than most global regions (Fig.2C).

**Fig.3:**
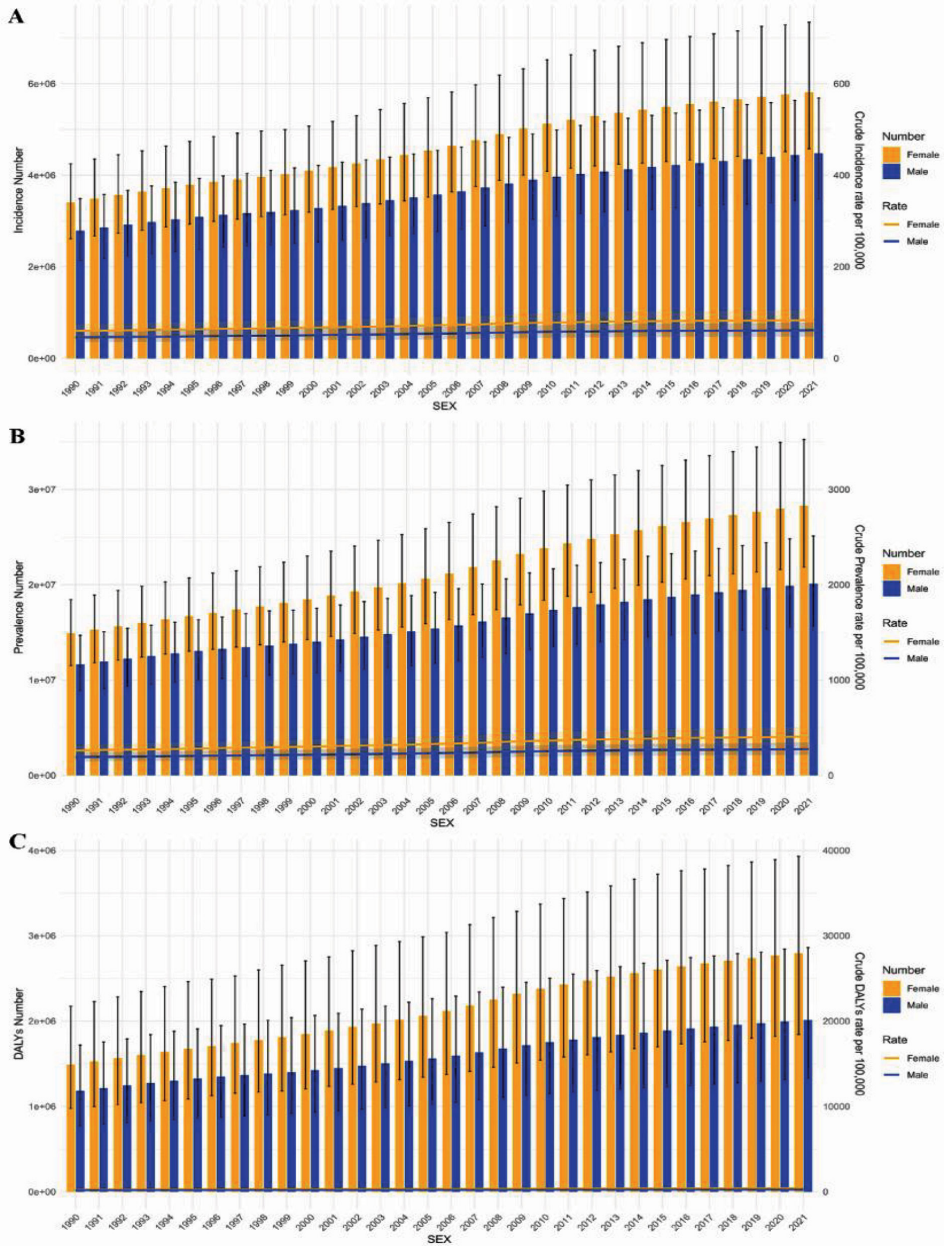
Temporal patterns of neck pain burden in China, 1990-2021. (A) ASIR; (B) ASPR; (C) Age-standardized DALYs rate.

**Fig.4:**
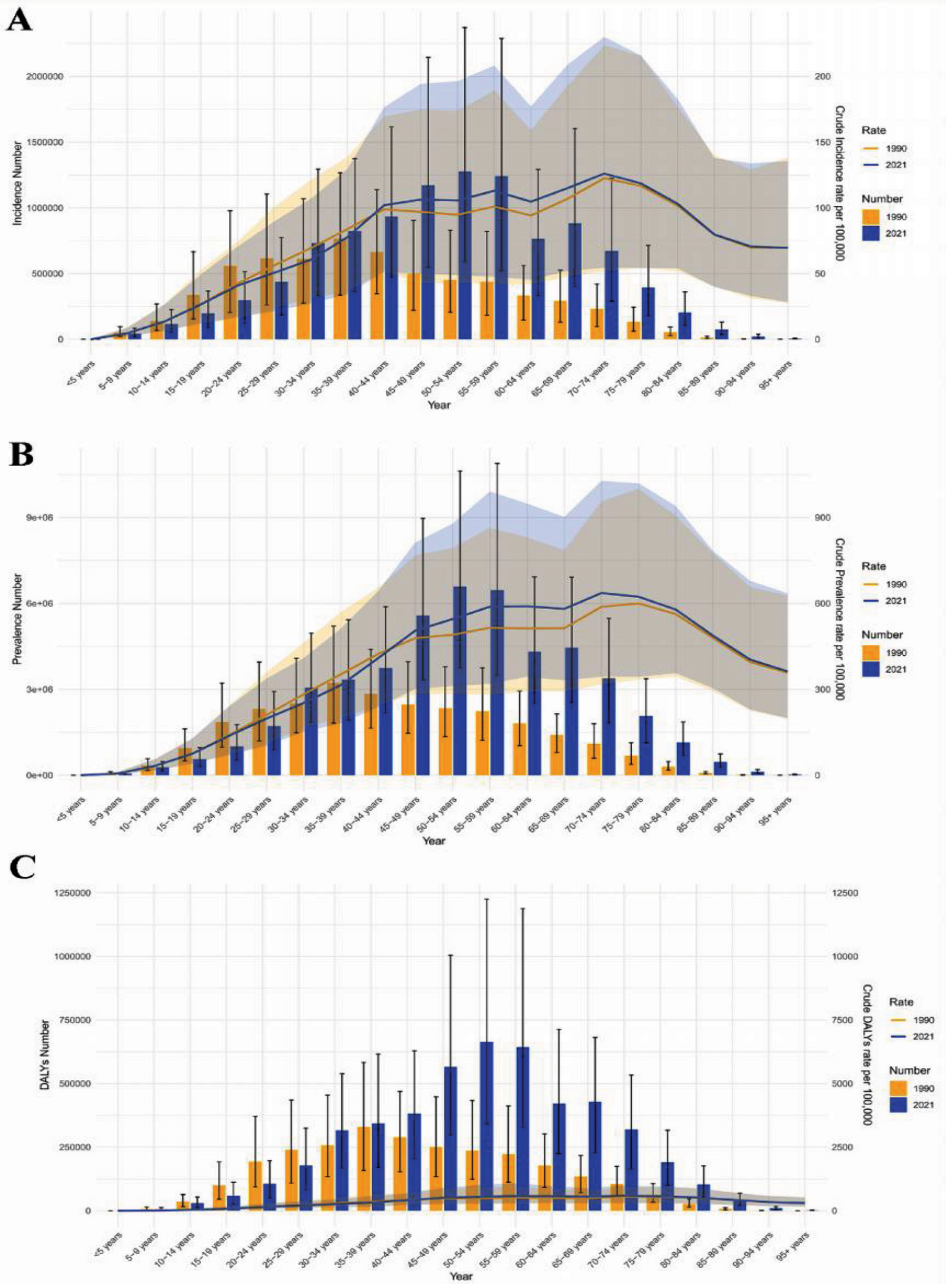
Age-specific burden of neck pain in China, 2021. (A) ASIR; (B) ASPR; (C) Age-standardized DALYs rate.

Between 1990 and 2021, all indicators of neck pain burden in China showed continuous increases, with females experiencing a higher burden than males, demonstrating apparent gender differences (Fig.3, Table-I). In 2021, the burden varied significantly across age groups: the incidence rate, prevalence rate, and DALYs rate initially rose with age, reaching a peak in the 70–74 age group before declining. In comparison, the total number of incidences, prevalence, and DALYs followed a similar pattern but peaked earlier, in the 50 – 54 age group (Fig.4). Although the overall age distribution of neck pain burden in 1990 resembled that of 2021, the peak age groups differed. In 1990, incidence rate, prevalence rate, and DALYs rate also peaked at 70 – 74 years, whereas total cases peaked at 35 – 39 years (Fig.4). These differences in peak age groups likely reflect the impacts of socioeconomic development, demographic shifts, and improvements in healthcare on the neck pain burden.

**Fig.5:**
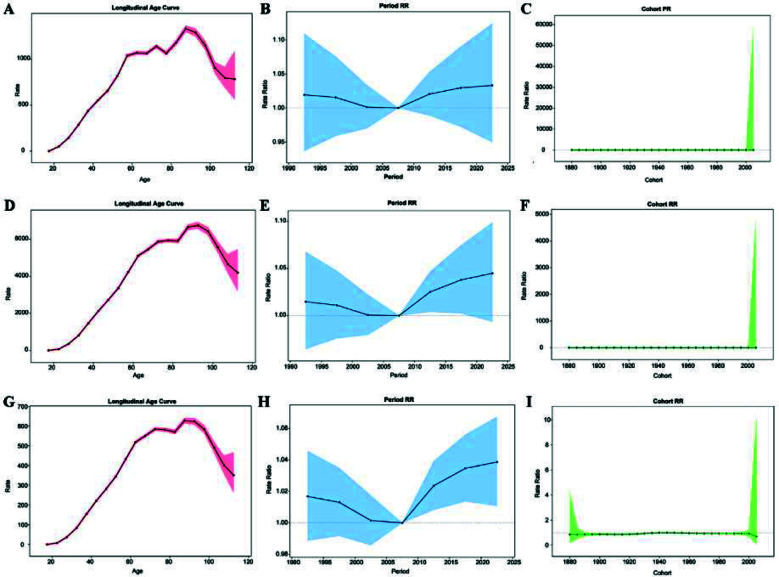
APC analysis of neck pain in China. (A) Age effect on incidence rate; (B) Period effect on incidence rate; (C) Birth cohort effect on incidence rate; (D) Age effect on prevalence rate; (E) Period effect on prevalence rate; (F) Birth cohort effect on prevalence rate; (G) Age effect on DALYs rate; (H) Period effect on DALYs rate; (I) Birth cohort effect on DALYs rate.

Analysis using the APC model indicated that the net drifts for incidence rate, prevalence rate, and DALYs rate of neck pain were 0.102% (95% CI: -5.516 – 6.054), 0.135% (95% CI: -9.898 – 11.285), and 0.123% (95% CI: 0.011 – 0.236) per year, respectively, indicating a gradual annual increase in the burden of neck pain (Table-II). Fig.5 demonstrates the estimated effects of age, period, and cohort on the incidence rate, prevalence rate, and DALYs rate derived from the APC model. The incidence rate, prevalence rate, and DALYs rate displayed age-dependent increases followed by decreases (Fig-5A, D, G). Regarding the period effect, the RR for incidence rate, prevalence rate, and DALYs rate decreased until 2005 – 2007, then progressively increased thereafter (Fig.5B, E, H). The cohort effect showed minimal variation, with RR values fluctuating around one for all three metrics (Fig.5C, F, I).

**Table-II:** Statistical parameters of neck pain in APC model.

Type	Net drift	P-value		
	(% per years 95% CI)	All local drifts = net drift	All cohort = deviations	All period = deviations
Incidence	0.102 (-5.516 – 6.054)	<0.001	<0.001	0.0089
Prevalence	0.135 (-9.898 – 11.285)	<0.001	<0.001	0.0007
DALYs	0.123 (0.011 – 0.236)	<0.001	<0.001	0.0005

**Fig.6:**
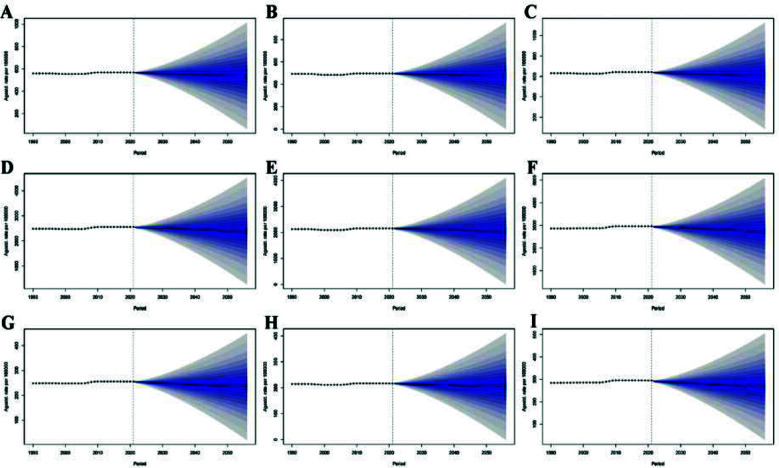
Projected trends in the burden of neck pain in China over the next 30 years. (A) Total ASIR; (B) Male ASIR; (C) Female ASIR; (D) Total ASPR; (E) Male ASPR; (F) Female ASPR; (G) Total age-standardized DALYs rate; (H) Male age-standardized DALYs rate; (I) Female age-standardized DALYs rate.

The BAPC model projections (Fig.6) indicated a significant shift in the future burden of neck pain in China from 2021 to 2050. The overall ASIR is estimated to decrease from 568 per 100,000 in 2021 to 542 per 100,000 in 2050 (Fig.6A), with similar downward trends observed in both males and females (Fig.6B, 6C). The ASPR is predicted to decline by 5.56%, falling from 2555 per 100,000 in 2021 to 2413 per 100,000 in 2050 (Fig.6D). In gender-specific terms, the male ASPR is expected to remain relatively stable, slightly decreasing from 2153 to 2067 per 100,000, while the female ASPR is projected to steadily decline from 2963 to 2771 per 100,000 (Fig.6E, 6F). Similarly, the age-standardized DALYs rate is forecasted to decrease overall, from 255 per 100,000 in 2021 to 237 per 100,000 in 2050, with reductions evident in both sexes (Fig.6G–I). Despite consistently lower indicator values in males across all three measures, the gender gap is expected to diminish over time, especially in prevalence.

## DISCUSSION

This study systematically analyzed the epidemiological characteristics and long-term trends of neck pain in China from 1990 to 2021 using data from the GBD 2021 study. Findings indicated that the ASIR, ASPR, and age-standardized DALYs rate for neck pain in China in 2021 were significantly higher than those observed in most regions and neighboring countries. All three indicators showed significant upward trends from 1990 to 2021, with females bearing a greater burden than males. Age-specific analysis revealed that incidence rate, prevalence rate, and DALY rate peaked in the 70 – 74 age group in 2021, whereas the absolute numbers of incidence, prevalence, and DALYs peaked earlier, in the 50 – 54 age group. These findings suggest that neck pain has become a significant public health problem in China.

Given the strong age dependency of neck pain, projected age-standardized rates (ASRs) are more informative of changes in underlying risk, as they largely remove the influence of population age structure and capture changes in age-specific rates. In contrast, projected absolute numbers and crude rates are jointly driven by population growth and population aging, and may remain high or increase even when ASRs decline. A formal decomposition analysis that partitions changes into population growth, population aging, and epidemiologic change in age-specific rates (e.g., Das Gupta’s method) would further quantify these contributions and strengthen the interpretation of projections.

The rising burden of neck pain in China is likely multifactorial. Population aging increases the proportion of individuals within high-risk age groups and elevates the prevalence of cervical degenerative diseases, thereby amplifying the overall burden.^1^,^2^,^12^ Consistent with our APC results showing that period effects declined until 2005–2007 and increased thereafter, this post-2005 increase should not be attributed to a single driver but may reflect multiple contemporaneous societal and health-system changes. Changes in lifestyle and occupational patterns, including greater sedentary time, prolonged screen/electronic device use, and reduced physical activity, may impose increased mechanical strain on the cervical spine, especially in urban populations.^2^,^11^,^20^,^21^ Meanwhile, expanded health insurance coverage and improved access to healthcare services since the mid-2000s may have increased recognition, documentation, and care-seeking for non-fatal musculoskeletal symptoms such as neck pain, potentially contributing to higher reported levels.^22^,^23^ In addition, rising health awareness and evolving perceptions of pain management may increase symptom reporting and healthcare utilization.^22^,^23^ Broader contextual factors such as heightened psychosocial stress, accelerated work pace, longer working hours, and increased workload may also contribute through pathways including sustained muscle tension, poorer sleep, and reduced physical activity.^9^ Nevertheless, community healthcare centers in China generally have limited capacity for early detection and intervention of neck pain, which may lead to treatment delays and suboptimal outcomes.^7^,^21^

Occupational exposures may play an important role in neck pain risk and may help interpret the observed age patterns.^1^,^2^,^24^–^26^ Sedentary, screen-based office work is often characterized by sustained forward-head posture, prolonged static neck/shoulder muscle loading, and repetitive keyboard or mouse tasks, which may promote cumulative strain and recurrent or chronic symptoms among working-age adults.^20^,^24^,^25^ In contrast, labor-intensive occupations more commonly involve heavy lifting, overhead work, repetitive bending or twisting, and vibration exposure, potentially increasing both acute injury risk and cumulative soft-tissue overload.^2^,^26^ Although GBD estimates are not stratified by occupation, changes in China’s occupational structure and longer working life may partly contribute to the relatively high absolute burden in middle-aged adults (around 50–54 years), while long-term cumulative exposure together with degenerative changes may contribute to higher rates in older ages (around 70–74 years).^3^,^12^ These hypotheses should be interpreted cautiously, as demographic shifts and healthcare-seeking or reporting patterns may also influence peak-age distributions.

As consistent with previous GBD studies on neck pain,^7^,^12^ the findings of this report indicate that the burden of neck pain is greater among females than males in China. This observation may be explained by several mechanisms, including gender-related roles and health-seeking behaviors.^27^ Additionally, traditional gender roles may also contribute to the increased prevalence of neck pain in the female population, as women are more frequently exposed to chronic strain from repetitive household tasks or the combined pressures of work and family responsibilities. Moreover, women may report pain symptoms and seek medical care more readily, potentially introducing gender bias in data, but also reflecting their greater health awareness.^28^

Beyond social and behavioral explanations, biological factors may also contribute to the higher burden observed among females. Declines in estrogen levels during the peri-menopausal and post-menopausal period could influence musculoskeletal structure and function, including muscle strength and endurance, tendon and ligament properties, and degenerative processes, and may affect pain processing and sensitivity, including susceptibility to central sensitization.^27^,^28^ In addition, sex differences in occupational and ergonomic exposures may be relevant; females may be overrepresented in jobs involving prolonged static postures, repetitive upper-limb tasks, or caregiving and service work with lifting and carrying demands, and unpaid domestic work may further increase cumulative musculoskeletal load.^2^,^26^ These mechanisms remain hypothesis-generating in the context of GBD-based analyses and warrant validation using individual-level data on hormonal status and occupational exposures.

The COVID-19 pandemic had a measurable impact on the neck pain burden. Between 2019 and 2021, both incidence and DALYs of neck pain in China increased slightly, significantly among females. This trend may be linked to widespread remote work, which promoted prolonged static postures, increasing cervical spine stress,^29^ diversion of medical resources toward the COVID-19 response, delaying diagnosis and treatment of other conditions, and elevated psychological stress, which can amplify chronic pain *via* central sensitization mechanisms.^30^

As neck pain is non-fatal, its DALY burden in the GBD framework mainly reflects years lived with disability (YLDs), which can translate into substantial indirect socioeconomic costs.^12^,^14^,^5^ Neck pain-related functional limitations may reduce work capacity and productivity through absenteeism and presenteeism, and may also increase healthcare utilization such as outpatient visits, imaging, medications, physical therapy, and rehabilitation.^1^,^2^,^5^ In addition, the relatively high absolute burden observed among middle-aged adults may disproportionately affect the core workforce and individuals with major family and caregiving responsibilities, amplifying broader social impacts.^2^,^5^,^21^ Persistent or recurrent symptoms may further increase long-term demand for rehabilitation and integrated pain management in primary care and chronic disease management systems.^1^,^2^,^21^

Our findings may inform practice-oriented priorities for neck pain management. In primary care, proactive case finding among older adults, particularly women, and working-age adults with persistent symptoms could support earlier identification. Initial stratification based on symptom duration, functional limitation, and red flags, such as neurological deficits or recent trauma, may facilitate timely referral and reduce chronicity. Because neck pain is often recurrent and non-fatal, home-based rehabilitation and self-management, including exercise-based neck/shoulder strengthening, posture and ergonomic adjustment, patient education, and adherence support, may help mitigate YLD burden when aligned with local clinical guidance.^31^,^32^ Targeted efforts for high-risk groups, including older women, could emphasize access to rehabilitation services, physical activity promotion, and follow-up to improve adherence.^12^,^31^,^32^ For the high-volume middle-aged workforce, workplace ergonomic programs, scheduled breaks, and related health promotion initiatives may be particularly relevant.^24^-^26^,^33^,^34^

The BAPC model in this study forecasted a declining trend in the burden of neck pain in China over the next 30 years, with a gradual reduction in gender differences. This projection corresponds with epidemiological patterns observed in some high-income countries, such as the United States and Europe,^33^ reflecting progress in health management, occupational safety, and healthcare accessibility in China. Despite the anticipated acceleration of population aging, the predicted decrease in disease burden suggests that aging is not the sole determinant. The period-effect analysis indicated a significant increase in neck pain burden since 2005–2007, which may be associated with widespread computer use, sedentary behavior, and increased smartphone use. Future reductions may result from targeted interventions, including the implementation of ergonomic equipment, adherence to cervical health guidelines, and enhanced early rehabilitation services. The study demonstrated that the cohort effect remains stable, suggesting minimal influence of birth cohort on neck pain risk. However, individuals born after 1990 may have distinct risk profiles, potentially characterized by higher education, improved health literacy, and greater engagement in health-promoting behaviors such as regular exercise, which may lower risk and contribute to a reduced future burden. Furthermore, advancements in artificial intelligence are expected to enhance prevention, diagnosis, and treatment strategies, further mitigating the neck pain burden over the coming decades.^32^,^35^

### Strengths:

The study leverages the latest standardized GBD 2021 estimates to provide an updated national assessment of neck pain burden in China from 1990 to 2021. We reported multiple burden metrics (incidence, prevalence, and DALYs) using age-standardized rates with 95% uncertainty intervals, and integrated complementary approaches (EAPC, APC, and BAPC) within one framework to quantify long-term trends, disentangle age–period–cohort patterns, and project trajectories to 2050. These age- and sex-specific characterizations help identify high-burden groups and enhance the public health relevance of the findings.

### Limitations:

This study is based on GBD 2021 estimates for a non-fatal condition, and several limitations should be considered. First, data availability and representativeness are uneven across time and locations, so GBD relies on statistical modeling and imputation using standardized Bayesian frameworks such as DisMod-MR 2.1. In China, mild-to-moderate neck pain is often self-managed and may be underdiagnosed or under-recorded in primary care and community settings, particularly in rural areas, which may lead to underestimation of the absolute burden. Regional heterogeneity in healthcare access, survey coverage, and recording systems may also introduce systematic reporting biases and increase uncertainty in national estimates and comparisons. Importantly, if under-ascertainment changes over time, observed temporal patterns and projections could be affected: improvements in detection and reporting may make apparent increases partly reflect better ascertainment rather than true risk increases, and the opposite could also occur. Second, measurement and definition differences may cause misclassification.

In GBD 2021, neck pain is defined using a standardized case definition, typically pain in the neck region lasting at least one month. Differences in survey instruments, recall periods, health-seeking behavior, and cultural perceptions of pain may influence symptom reporting and contribute to measurement error and recall bias. Third, estimates depend on modeling assumptions and uncertainty propagation across multiple GBD components. For neck pain, DALYs largely reflect YLDs, which are jointly determined by prevalence, severity distributions, and disability weights; uncertainties and assumptions in these components can propagate and potentially accumulate along the estimation chain. In addition, APC period effects represent population-level temporal signals and do not isolate specific causal drivers, so related interpretations should be regarded as contextual and hypothesis-generating.

Our BAPC projections to 2050 assume continuation of historical patterns; structural changes in healthcare-seeking behavior or data quality could shift future trajectories. To enhance transparency and comparability, we emphasized age-standardized rates and 95% uncertainty intervals, but these steps cannot fully eliminate bias arising from under-ascertainment, regional heterogeneity, or model dependence. Projections should therefore be interpreted together with demographic context. Finally, scope-related constraints remain. GBD outputs do not provide occupation-stratified estimates and do not include individual-level information on hormonal status or symptom course and recurrence (acute/subacute/chronic), which prevents sex-specific biological analyses as well as occupation- and course-specific analyses in this study. We also did not conduct a formal decomposition analysis to quantify the relative contributions of population growth, population aging, and changes in age-specific rates to projected burden. Moreover, productivity losses and direct/indirect economic costs could not be quantified because such information is not available in GBD estimates. Accordingly, the clinical implications discussed should be interpreted as population-level considerations rather than patient-level recommendations.

### Suggestions:

To improve data accuracy and strengthen China-specific evidence beyond aggregated GBD estimates, future research should implement nationwide, neck pain-specific epidemiological surveys and integrate health records with data from wearable devices or smartphone sensors to capture incidence and care-seeking more comprehensively. Prospective cohorts and real-world studies across regions and occupational groups are also needed, with detailed phenotyping of symptom duration and recurrence (acute/subacute/chronic), functional outcomes, imaging findings, and treatment response.

These designs would enable more rigorous evaluation of key behavioral and ergonomic risk factors, including occupational exposures (sitting duration, physical workload, vibration, and ergonomics) and screen time, and would support occupation-stratified analyses by linking GBD-based estimates with national occupational classifications, labor force or health surveys, cohort data, and administrative datasets. In parallel, more flexible dynamic prediction approaches that incorporate socioeconomic factors, public health policies, and indicators of emerging technologies may complement long-horizon BAPC projections by enabling finer-grained forecasting and risk stratification. Causal inference strategies, including Mendelian randomization where feasible, could help clarify causal relationships between candidate risk factors and the incidence and progression of neck pain, and may also shed light on biological contributors to sex disparities.

In addition, decomposition analyses that separate the effects of population growth, population aging, and changes in age-specific rates (such as Das Gupta’s three-component method) would strengthen the policy interpretability of projections. Finally, cohort- or survey-based classifications by symptom duration and recurrence could help determine whether chronic or recurrent cases disproportionately drive increases in years lived with disability.

## CONCLUSION

This study provides an updated assessment of the burden of neck pain in China from 1990 to 2021 using GBD 2021 estimates and projects patterns to 2050. The burden remains substantial, with higher rates among females and older adults and a high absolute burden among middle-aged populations. Projections suggest that age-standardized rates may decline, but population growth and aging may keep the absolute burden high. Given uncertainties inherent in GBD-based estimation and modeling, findings should be interpreted with attention to 95% uncertainty intervals (UIs). Strengthening early identification and integrated rehabilitation in primary care, scalable home-based self-management, and workplace ergonomic interventions—especially for high-risk groups such as older women—may help mitigate future health and socioeconomic impacts.

### Authors’ contributions:

**GZ:** Literature search, study design and manuscript writing. **Xian Zhang, Xinrui Zhao, SZ and HZ:** Data collection, data analysis and interpretation. Critical review. **GZ:** Manuscript revision and validation and is responsible for the integrity of the study. All authors have read and approved the final manuscript.
